# PHN List of Reviewers

**DOI:** 10.1017/S1368980023000162

**Published:** 2023-04

The Editorial Board of *Public Health Nutrition* would like to thank the following for their contribution as peer reviewers in 2022:

Julie Aarestrup

Leila Abar

Joana Abou-Rizk

Taymara Abreu

Mohamed Abuelazm

Magaly Aceves-Martins

Rachel Acton

Ishag Adam

Robert Adams

Folasade Adebayo

Tulsi Adhikari

Laura Adubra

Brooke Aggarwal

George Agogo

Aryati Ahmad

Arif Ahmed

Karar Zunaid Ahsan

Muhammad Sajid Hamid Akash

Blessing Akombi-Inyang

Robert Akparibo

Katherine Alaimo

Janice Albert

Nawal Al-Qaoud

Naser Alsharairi

Emily Altman

Carlos Alvarez Dardet Diaz

Mariane Alves

Muzna Fatima Alvi

Ramya Ambikapathi

Sarah Amin

Pedro Andrade

Tatiana Andreyeva

Mitravinda Aneesh

Daniel Antiporta

Katherine Appleton

Sandra Arana

Chrisa Arcan

JoAnne Arcand

Frank Ardesch

Katie Arlinghaus

Francis Arthur-Holmes

Mahmoud Askari

Vincent Assey

Amy Auchincloss

Brekhna Aurangzeb

Catarina Azeredo

Mina Babashahi

Laxman Bablani

Shayan Bahadori

Marcia Baldisserotto

Olukunmi O. Balogun

Michela Balsamo

Khor Ban Hock

Daniel Bandoni

Larissa Baraldi

Thomas Baranowski

Josep Xavier Barber Valles

Liza Barbour

Alan W. Barclay

Helga Bárdos

Carolina Bassul

Mônica Batalha

Sarah Bath

Danielle Battram

Kaleab Baye

Alyssa Beavers

Emma Beckett

Elodie Becquey

Rebecca Beeken

Alicia Beltran

Sara Benjamin Neelon

Christie Bennett

Lisa Bero

Ronel Beukes

Ilana Nogueira Bezerra

Komal Bhatia

Dharma Bhatta

Ghose Bishwajit

Peter Bjerregaard

Jennifer Black

Maureen Black

Rachel Bleiweiss-Sande

Leigh Blizzard

Anna Bogacka

JN Bonglaisin

Sue Booth

Kaushik Bose

Lais Botelho

Emma Bouman

Vasiliki Bountziouka

Bernard Brabin

Caroline Brand

Sarah Breathnach

André Briend

Flávia dos Santos Brito

Amy Brown

Leanne Brown

Julia de Bruyn

Opal Buchthal

Nassib Bueno

Aurora Bueno-Cavanillas

Luciene Burlandy

Rebecca Byrne

Felix Caballero

Leah Cahill

Juliana Camargo

Elizabeth Campbell

Daniela Canella

Nuray Caner

Jonathan Cantor

Raquel Canuto

Giovanna Caparello

Julia Carins

Monica Carlsen

Valeria Carpio-Arias

Angela Carriedo

Thais Carrilho

Alicia Carriquiry

Jennifer Carter

Pedro Carvalho

Anna Casey

Caitlin Caspi

Omni Cassidy

Laura Lara Castor

Paulo Cesar Castro Junior

Bess Caswell

Monica Cattafesta

Laura Caulfield

Gustavo Cediel

Anina Chain

Stephanie Chambers

Catherine Chan

Te-Fu Chan

Jack Chapel

Karen Charlton

Helene Charreire

Jorge Chavarro

Liam Chawner

Nuzhat Choudhury

Benjamin Chrisinger

Meaghan Sarah Christian

Marta Citelli

Emmanuel Cohen

Juliana Cohen

Pieter Cohen

Cynthia K. Colapinto

Renee Cole

David Colozza

Kirsten Coppell

Clare Corish

Laura Cornelsen

Teresa Correa

Daniel Corsi

Bruna Costa

Caree Cotwright

Gundersen Craig

Carlos Cruz Casarrubias

Idiko Csolle

Katherine Cullerton

Ana Paula Cupertino

Cintia Curioni

Zhaoli Dai

Fidelia Dake

Jane Dancey

Shaonong Dang

Heather D'Angelo

Muhammad Arif Darmawan

Subhasish Das

Rajat Das Gupta

Mike Daube

Jaimie Davis

Kathleen Davis

Camila de Carvalho

Blandine de Lauzon-Guillain

Milena de Moraes

Alessandra de Oliveira

Amila de Silva

Marian de van der Schueren

Nanne de Vries

Kirk Dearden

Paulien Decorte

Gauri Desai

Mulugeta Desalign

Melanie Deschasaux

Emily Dewitt

Jigna Dharod

Jennifer Di Noia

Lauren Dinour

Bernice Dodor

Marcela Dofkova

Radhouene Doggui

David Doledec

Kimberly Dong

Yanhui Dong

Shufa Du

Lisanne Du Plessis

Eleanor Dunlop

Samuel Duran

Jennifer Dush

Pratibha Dwarkanath

Johanna Dwyer

Creswell Eastman

Gudina Egata

Heather Eicher-Miller

Danielle L. Eiseman

Nassim El Achi

Tatiana El-Bacha

Basma Ellahi

Charlene Elliott

Morgan Ellithorpe

Pauline Emmett

Carla Enes

Rachel Engler-Stringer

Mihela Erjavec

Maijaliisa Erkkola

Getahun Ersino

Günay Eskici

Stephanie Ettinger de Cuba

Seth Etuah

Patricia Eustachio Colombo

Shiva Faghih

Jennifer Falbe

Somaye Fatahi

Judhiastuty Februhartanty

Beth Feingold

Ana Feldenheimer da Silva

Fentaw Wassie Feleke

Marcos Ferasso

Aline Ferreira

Antonio Augusto Ferreira Carioca

Giovanna Fiates

Roger Figueroa

Pilar Flores

Karen Florez

Maryah Fram

Deborah Frank

Sarah Frank

Gary Fraser

Stephane Frayon

Nathalia Freitas-Costa

Edward Frongillo

Aya Fujiwara

Kamila Gabe

Sheila Gahagan

Jamie Gahche

Artur Galimov

Danielle Gallegos

Sina Gallo

Patricia Galvez

Navika Gangrade

Kyle Ganson

Elsa Gaona-Pineda

Ada Garcia

Karin Geffert

Duke Gekonge

Aulo Gelli

Michael Georgoulis

Sarah Gerritsen

Derje Getahun

Heather Gibbs

Alice Gibson

Rachel Gillespie

Jason Gilliland

Konstantinos Gkiouras

Benoit Gnonlonfin

Justyna Godos

Mahdieh Golzarand

Caroline Gomes

Fabio Gomes

Clara Gómez-Donoso

Fabiana Gonçalves

Sabrina Ionata Granheim

Antoneta Granic

Eva Greenthal

Ted Greiner

Jessica Grieger

Mariana Grilo

Piyush Gupta

Alison Gustafson

Joanne Guthrie

Roger Gutierrez-Juarez

Kyungho Ha

Maria Claret Hadler

Luc L. Hagenaars

Robert Hamlin

Polly Hardy-Johnson

Brook E. Harmon

Jessica Harris

Ashleigh Hart

Isabella Hartley

Christina Hartmann

Rebecca Hasdell

Andreas Hasman

Jennifer Hatchard

Sandi Hayes

Ashleigh Haynes

Mary O. Hearst

Valisa Hedrick

Samantha Heerschop

Rebecca Heidkamp

Erin Hennessy

Maria Henström

Jacqueline Hernadez

Bernardo Hernandez

Roland Herrmann

Isabelle Herter-Aeberli

Jillian Hill

Frances Hillier-Brown

Matthew Hobbs

Erin Hobin

John Hoddinott

Jody Hoenink

Catherine Holtmann

Reza Homayounfar

Graham Horgan

Katy Horner

Paula Horta

Susan Horton

Freddy Houngbe

Dongsheng Hu

Liping (Polly) Huang

Jorg Huber

Clare Hughes

Oliver Huse

Misba Hussain

Scott Ickes

Iris Iglesia

Eri Imai

Elif Inan-Eroglu

Ana Irache

Leila Itani

Suvi Itkonen

Kia Nöhr Iversen

Toshiyuki Iwahori

Jana Jabbour

Paul Jacques

Edward Jaenicke

Alireza Jahan Mihan

Patricia Jaime

Zeina Jamaluddine

Kathryn Janda

Megan Jarman

Melissa Jensen

Theresa Jeremias

Mahsa Jessri

Jenny Jia

Kari Johansson

Brittany Johnson

Rebecca Jones-Antwi

G Kenđel Jovanović

Juliana Kain

Yuya Kakutani

Nipa Kamdar

Nena Karavasiloglou

Sharon Karp

Noora Kartiosuo

Tilakavati Karupaiah

Suna Kassier

Michelle W. Katzow

Festo Kavishe

Justine Kavle

Dirghayu KC

Bridget Kelly

Erica L. Kenney

Monique Potvin Kent

Marko Kerac

Mathilde Kersting

Preeti Khanna

Maryam Khojasteh

Kijoon Kim

Kirang Kim

Aygül Kissal

Carmen Klinger

Frances Knight

Linda Knol

Eda Koksal

Joel Komakech

Amanda Kopetsky

Liisa Korkalo

Emma Kortekangas

Vivica Kraak

Julia Krasevec

Frank Kraus

Michael Krawinkel

Jaclyn Kropp

Mukesh Kumar

Tony Kuo

Amos Laar

Elisa Maria Lacerda

Hanna Lagtström

Gurpinder Lalli

Fernando Lamarca

Abera Lambebo

Jessica G. LaRose

Nicole Larson

Gwenyth Lee

Jiwoo Lee

Rebecca Leech

Maria Alvim Leite

Alessandro Leone

Lucia Leone

Jef Leroy

Meron Lewis

Kelin Li

Yan Li

Bojing Liao

Harris Lieberman

Djin Gie Liem

Angela Liese

Rebecca Lindberg

Anna Karin Lindroos

Anna Lipert

Cosima Lisi

Natalie Lister

April Litchford

Fiona Lithander

Zhengping Liu

Lindsey Locks

Ivory Loh

Pinpin Long

Noemi Lopez-Ejeda

Esther Lopez-Garcia

Philip Loring

Jimmy Chun Yu Louie

Barbara Lourenco

Penny Love

Anne Løvhaug

Hui Luan

Nyati Lukhanyo

Jacqueline Lyons

Gary Ma

Guansheng Ma

Lesley MacDonald-Wicks

Priscila Pereira Machado

Una MacIntyre

Sally Mackay

Hazreen Majid

Miltiadis Makrygiannakis

Kenneth Maleta

Vasanti S. Malik

Md. Maniruzzaman

Liina Mansukoski

Muchtaruddin Mansyur

Aline Mariath

Grace Marquis

Stephanie Martin

Ana Paula Martins

Kate Mason

Travis Masterson

Saeed Mastour Alshahrani

Roger Mathisen

Louisa Matwiejczyk

Chelsea Mauch

Mohsen Mazidi

Teresia Mbogori

Amanda McClain

Judith McCool

Lacey McCormack

Christine McDonald

Marie McGrath

Lynn McIntyre

Rachael McLean

Taylor McLinden

Fernanda Mediano

Yohannes Melaku

Fei Men

Larissa Mendes

Xiang-Yu Meng

Daniel Miller

Lisa Miller

Diane Mitchell

Kyoko Miura

Michel Mocellin

Mahmoud Mohammad

Noushin Mohammadifard

Itismita Mohanty

Zalilah Mohd Shariff

Ali Montazeri

Luana Monteiro

Rob Moodie

Elsie Moore

Sophie Moore

Maria Morales Suárez-Varela

Mercedes Mora-Plazas

Judy More

Belinda Morley

Jean-Claude Moubarac

Sofia Mouchti

Claire Mouquet-Rivier

Hayet Moussa

Dariush Mozaffarian

Rebecca Mozaffarian

Gudani Mukoma

Joshua Muliira

Mary Murimi

José Muros

Blain Murphy

Rachel Murphy

Nandita Murukutla

Antonina Mutoro

Martha Mwangome

Candice Myers

Joanna Myszkowska-Ryciak

Muzi Na

Sayaka Nagao-Sato

Mieko Nakamura

Nathalia Naspolini

Lara Nasreddine

Elizabeth Neale

Cindy Needham

Jennifer Nelson

Marion Nestle

Merryn Netting

Shu Wen Ng

Yandisa Ngqangashe

Kim Nguyen

Mary Nicolaou

Claudia Nieto

Eduardo Nilson

Nobuo Nishi

Jennifer Norman

Tanita Northcott

Kate Northstone

Kieran O'Brien

Lauren O'Connor

Veronica Ojiambo

Kufre Okop

Frank Okwan

Beatrice Olack

Catherine Oldenburg

Mayara Oliveira

Stephen Onufrak

Colin J. Orr

Alejandro Ortega

Naiá Ortelan

Aaron J. O'Sullivan

Rei Otsuka

Ifeoma Ozodiegwu

Helena Pachon

Valeria Pala

Claire Palermo

Poliana Palmeira

Sherly Parackal

Kyong Park

Jennie Parnham

Stephanie Partridge

Anisha Patel

Elise Pauzé

Natalie Pearson

Meredith Peddie

Lucia de Fátima Pedrosa

Dolores Penafiel

Jiahao Peng

Tarra Penney

Mary Penny

Catherine Pereira

Kingsley Pereko

Ines Perrar

Henry Perry

Fanny Perterman-Rocha

Joshua Petimar

John Pettifor

Simone Pettigrew

Payao Phonsuk

Adriano Pimenta

Luigi Piper

Marta Plichta

Ana Poblacion

Catherine Pollack

Jennifer Pomeranz

Mary Kathryn Poole

Hannah Posluszny

Makan Pourmasoumi

Thomas Power

Iain S. Pratt

Igor Pravst

Melissa Prescott

Rachel Prowse

Seema Puri

Sven Putnis

Amm Quamruzzaman

Anna Queiroz de Medeiros

Yara Qutteina

Maja Radojčić

Berna Rahi

M. Shafiqur Rahman

Md. Rahman

Sabuktagin Rahman

Hasina Rakotomanana

Prof Kaushik Ramaiya

Maria J. Ramirez-Luzuriaga

Anu Rammohan

Neha Rathi

Kethmany Ratsavong

Fernanda Rauber

Jofrey Raymond

Shaharior Rahman Razu

Erica Reeve

Marla Reicks

Gláubia Relvas

Dan Remley

Yanjun Ren

Brandon J. Restrepo

Marcela Reyes

Cecília Cláudia Ribeiro

Scott Richardson

Malcolm Riley

Natascia Rinaldo

Lauren A. Roach

Rebecca Robert

Eric Robinson

Brenda Robles

Pamela Robles-Valcarcel

Shannon Robson

Rachel Rodgers

Michele Rodrigues

Alexander Rodriguez

Sonia Rodriguez-Ramirez

Federico Roncarolo

Eva Roos

Donald Rose

Airin Roshita

Asher Rosinger

Lucila Rozas

Maria Rubin-Garcia

Pasquale Rummo

Cherie Russell

Joanna Russell

Petra Rust

Suzanne Ryan-Ibarra

Joachim Sackey

Richard Sadler

Janine Saga

Mefkure Sahin

Nadine Sahyoun

Aki Saito

Amin Salehi-Abargouei

Rosana Salles-Costa

Laura J. Samuel

Emma Sanchez-Vaznaugh

Tina Sanghvi

Jacob Sarfo

Haribondhu Sarma

Hugo Sarmento

Daniela Sartorelli

Isabela Sattamini

Karoline Sauder

Cláudia Saunders

Tailane Scapin

Roberto Scaramuzzino

Ellen Schafer

Raquel Schincaglia

Sabrina Schlesinger

Ashley Schram

Peter Schulz

Tracy Schumacher

Sara Schwanke Khilji

Marlene Schwartz

Rebecca Schwei

Stephanie Scott

Maree Scully

Aidan Searle

Paraskevi Seferidi

Hilary Seligman

Danit Shahar

Xianwen Shang

Meghan M. Shea

Richard Sheward

Meghan Shirley

Eleanor Shonkoff

Sangeetha Shyam

Rosely Sichieri

Madeleine Sigman-Grant

Natanael Silva

Priscila Silva

Wanderson Silva

Angelie Singh

Som Singh

Chelsea Singleton

Jordana Siqueira

Binyam Sisay

Susan Sisson

Guri Skeie

Sinne Smed

Claire Smith

Dianna Smith

Kiah Smith

Matthew R. Smith

Cornelius M. Smuts

Marta Solans

Jessica Soldavini

Mark Soto

Fabiola Souza

Juliana Souza

Suzanne Spence

Angela Spinelli

Chittur Srinivasan

Denes Stefler

Steen Stender

Marinka Steur

Cristina Stewart

Nelia Steyn

Barbara Stoecker

Pasquale Strazzullo

Josine Stuber

Roland Sturm

Christopher Sudfeld

Minami Sugimoto

Guiju Sun

Jennifer Tabler

Wakako Takeda

Alison Talbert

Xueying Tang

Menghua Tao

Linda Tapsell

Valerie Tarasuk

Ayten A. Tas

Mimi Tatlow-Golden

Sharon Taverno Ross

Rachael Taylor

Nicolette Teufel-Shone

Tinku Thomas

Lukar Thornton

Anne Marie Thow

Faten Tlili

Ulla Toft

Andrew Tomita

Susan Torres

Zoi Toumpakari

Alison Tovar

İsmail Toygar

Kathy Trieu

Aron Troen

Angela C. B. Trude

Tilahun Degu Tsega

Margarita D. Tsiros

Shobha Udipi

Ken Uechi

Desiree Valera-Gran

Andréia Valim

Jan Willem van der Kamp

Tabitha van Immerzeel

Ellen van Kleef

Tertia van Zyl

Lana Vanderlee

Carmen Vargas

Hassan Vatanparast

Juliana Vaz

Gabriela M. Vedovato

Ines Velasco

Sudershan Vemula

Sonia Venancio

Sudha Venkatramanan

Carina Venter

Henna Vepsäläinen

Letícia Vertulli

Parisa Vidafar

Rodolphe Vidal

Helen Vidgen

Regina Coeli Vieira

Florent Vieux

Frøydis Vik

María Fernanda Vinueza-Veloz

Fernando Vio

Marianne Visser

Marina Visser

Marcia Vitolo

Antonis Vlassopoulos

Rachel Vollmer

Sebastian Vollmer

Peter von Philipsborn

Wieger Voskuijl

Katharina Wabnitz

Maria Wakolbinger

Corinna Walsh

Christine Walters

Karen Walton

Xuemei Wang

Lisa Ware

Sarah Warkentin

Judith Waswa

William Waters

Daniella Watson

Wendy Watson

Lorraine Weatherspoon

Qiang Wei

M. Margaret Weigel

Nancy Weinfield

Jonathan Wells

Megan Whatnall

Mariaan Wicks

Michael Widener

Kurt Widhalm

Simon Wieser

Parke Wilde

Anne Williams

Renate Winkels

Julia Wolfson

Beverly Wolpert

Jyh Eiin Wong

Alexander Wray

Charlotte Wright

Stephanie Wrottesley

Di Wu

Wei-Te Wu

Fei Xu

Amit Yadav

Tuba Yalçın

Alice Yan

Nianhong Yang

Wenjie Yang

Yi Yang

Sevil Yılmaz

Heng Yaw Yong

Cynthia Yoon

Serene Yoong

Leanne Young

C.Q. Yu

Barakatun-Nisak Yusof

Francesca Zambri

Antonis Zampelas

Farid Zayeri

Wanga Zembe-Mkabile

Dongfeng Zhang

Jianduan Zhang

Zhao Zhao

Weiwei Zheng

Yuqing Zheng

Mi Zhou

Ming Zhou

Qianling Zhou

Meghan Zimmer

Christina Zorbas

Zhi-Yong Zou

Adama Zoubga

